# What Means Does the Modern Obstetrician Employ to Prevent Ophthalmia of the Newly Born?

**Published:** 1905-10

**Authors:** J. Clifton Edgar

**Affiliations:** New York


### What Means Does the Modern Obstetrician Employ to Prevent Ophthalmia of the Newly Born? [2]

By J. Clifton Edgar, M. D., of New York.

[*Medical News*, September 25, 1905]

IT goes without saying that no measure should be neglected which can assist in preventing the occurence of ophthalmia neonatorum. From the standpoint of the obstetrician there are two means at our command for reducing in frequency, if not practically preventing, ophthalmia neonatorum. These are: First, the ante-partum preparation of the maternal passages in cases in which they are suspected of infection; second, the dropping into each conjunctival sac immediately after delivery of some antiseptic, as nitrate of silver, protargol or argyrol.

When the maternal passages are suspected, a preliminary course of treatment of the vagina should be instituted, beginning about two weeks before delivery. This should consist in daily or twice daily vaginal douching, first with a mild alkaline solution and then with one of bichloride of mercury in strength 1-5,000. Just before delivery something must be done to provide a substitute for the normal lubricating mucus which will have been washed away by the douching process, and a one per cent. lysol solution will be found useful for this purpose. The vagina may be washed out with it when labor begins.

The scientific gospel of Bumm and Krönig, namely, that the vaginal mucus has a bactericidal action, has been misunderstood as applying to gonococcus infection. It has no such action. Today there is abundant evidence from many sources to prove that the fetus is in certain cases infected with the gonococcus *in utero* and hence born with gonococcus infection of the conjunctiva. Thus no prophylaxis either in the nature of ante-partum vaginal cleansing or post-partum instillation into the conjunctival sac of antiseptics can in a small percentage avail anything.

If the vagina, as many today believe, is usually sterile, it needs no preparation for labor to prevent ophthalmia, if the reverse is true, it does.

At the Emergency Bellevue service and at the New York Maternity, after repeated experiments with and without ante-partum, antiseptic preparation of the vagina for labor, I have finally been compelled to use it in all cases without exception, in order to keep my morbidity rate from ophthalmia neonatorum at the lowest figure. Both of these services, however, are largely venereal in character.

At the Manhattan Maternity and Dispensary I am now making use of antiseptic preparation of the vagina in selected cases only, but as the service is at present only three months old we can report nothing of value. In this time we have had one case of ophthalmia neonatorum, and that in spite of Credé's post-partum treatment.

In private practice I am not accustomed to use ante-partum vaginal cleansing—this being a concession to the popular belief that there is less gonococcus infection in private practice, and yet every now and then I see gonococcus infection in spite of Credé's nitrate of silver method of prevention. I had such a case some years ago in my practice, where the husband had his acute gonorrhea in August and married in January. The baby all but lost the sight of both eyes, but finally recovered.

I have every faith in Credé's method for the prevention of ophthalmia neonatorum. In both hospital and private cases I am now accustomed to make use of this method without exception. After repeated experiments with protargol I have found nothing to compare with silver nitrate in 2 per cent. solution as a prophylactic. As soon as the child is born its face is carefully washed with boric acid solution, seperate wipes being used for each eye, and the lids rubbed from the nose outward in each case. Then whether infection is suspected or not, two drops of a 2 per cent. solution of silver nitrate are dropped into each conjunctival sac. The excess is washed away in about thirty seconds with a normal saline solution.

In my experience, solutions of less than 2 per cent. do not destroy bacteria, and although solutions of 3 per cent. are harmless, they are apt to cause "silver catarrh," and are not required.

Of the many substitutes recommended it is very difficult to determine whether they are equal to or have any superiority over Credé's 2 per cent. nitrate of silver, but in my experience, after experimentation in hospital practice, protargol and argyrol are not equal in efficiency to the nitrate of silver treatment.

Ante-partum vaginal cleansing in suspected cases and Credé's nitrate of silver method after birth cannot absolutely abolish gonorrheal ophthalmia, because of the small percentage of intra-uterine gonococcus infection, but they can reduce the morbidity to practically *nil.* As long as men with gonorrhea are permitted to marry and women with gonoccus infection to conceive, so long will there be danger of gonorrheal ophthalmia in the newly born child.

My faith in the prophylactic power of the nitrate of silver method is so strong that I attribute all apparent negative or ill effects of the method to the presence of ante-partum infection of the eyes, to unskilful application or to improper or inert solutions.

Dauber[1] states that antagonism to Credé's method has developed in quite recent years. First, it has been shown that the method has not been carried out properly—this, of course, being no fault of the method itself; still, as gonorrheal blindness has only been reduced less than one-half, something is wrong. Midwives should be compelled to use it. The second objection is that Credé's method tends to increase eye morbidity by causing "silver catarrh." This has led to the use of argyrol and protargol. Zweifel uses the former, and neutralises with sodium chloride. His morbidity in over 5,000 cases has been but 0.23 per cent. Scipiades finds argyrol a certain prophylactic. From a number of other clinics come equally good reports as to protargol.

Recently in Hofmeier's clinic Credé's solution has been reduced from two per cent. to one per cent., following the custom of Runge, Fehling and Gusserow. Since this reduction the morbidity in over 5,000 cases has been but 0.33 per cent., with no silver catarrh worth mentioning. Thus while there was no dissatisfaction with Credé's method, the author concluded to give argyrol and protargol a suitable trial. Not only were the results less favorable, but more silver catarrh was caused by Zweifel's method than by any other single procedure. Hence, at the Hofmeier clinic, Credé's one per cent. is regarded as the best prophylactic. Hofmeier also uses an ante-partum douche in his clinic, which is naturally prophylactic to ophthalmia neonatorum. Incidentally it is stated that the morbidity of this condition varied in the past with that of sepsis. When puerperal fever used to be epidemic, there was an eye morbidity of 50 per cent. at times. General asepsis, etc., brought this down to 10-12 per cent., while Credé's method has almost obliterated morbidity of the eye in individual clinics.

Wintersteiner[1] analyzes 122 cases of actual ophthalmia neonatorum. Two were undoubtedly cases of ante-partum infection. In forty others the disease did not develop until after the fifth day, and at various intervals, hence regarded as possibly of post-partum infection, although some authorities believe in delayed intra-partum causation, due to weakened virulence of germs. Credé's method is of great value within certain limits, but cannot prevail against anomalous cases, such as those of late development. To include the latter, whatever the causes, prophylaxis must be kept up through puerperium.

Wintersteiner uses Stellwag's method—potass. permanganate douching (1-1,000) at short intervals, with Credé's solution twice daily. In his series of cases he obtained complete cures in every instance.

Alvarado[2] has been an industrious compiler of statistics, but does not always give the clinics. In one series of 6,397 cases a single drop of Credé was instilled and not an infection followed. In a large series of over 15,000 cases about two per cent. developed the disease in spite of Credé's method. Other clinics go still higher (up to five per cent.). However, in clinics in which the solution used was but 1.5 per cent., the morbidity was much greater, running as high as 7.5 per cent., and even 12.2 per cent. As the author is a Spaniard, many if not all of his figures are probably of local origin. As for protargol, the only large figures he knows of are those of Rubesca, of Prague (morbidity of two cases in 1,100). These read well, but what are they in comparison with the old records of Leopold (3,000 cases of Credé's method without any morbidity)!

*Does Credé's Method Cause Conjunctivitis?*—Neither Leopold (in 3,000 cases) nor Rosthorn (in nearly 25,000 cases) ever witnessed this accident. Author cites Bischof's view that conjunctivitis never results if technic has been correct.

In regard to treatment, Alvarado sent out a circular of inquiry to colleagues (oculists) in all parts of the world as to relative merits of Credé's method on one hand and the substitution of *protargal,* on the other. He received 31 replies, all but one in favor of Credé's. The exception was the Lyons clinic, where protargol is in use.

Ernst[1] discusses Credé's method very briefly with reference to the conjunctivitis which it is said to cause. No gonococci are ever found in these cases. They are due therefore to the nitrate. He experimented with a 1.5 per cent. solution and still got the conjunctivitis. Finally he found that the one per cent. solutions were non-irritant. At the Cologne Maternity, from January 1, 1902, to July 1, 1904, this author employed the one per cent. solution in 1,111 births. Not a single case of ophthalmia developed.

